# High-resolution imaging enabled by 100-kW-peak-power parametric source at 5.7 THz

**DOI:** 10.1038/s41598-023-32969-8

**Published:** 2023-04-10

**Authors:** Cang-He Kuo, Ming-Hsiung Wu, Chieh-Ru Chen, Yan-Jou Lin, Fredrik Laurell, Yen-Chieh Huang

**Affiliations:** 1 Department of Electrical Engineering, Institute of Photonics Technologies, National Tsing Hua University, Hsinchu, 30013 Taiwan; 2 Department of Applied Physics, Royal Institute of Technology, Roslagstullsbacken 21, 10691 Stockholm, Sweden

**Keywords:** Nonlinear optics, Terahertz optics, Imaging and sensing

## Abstract

Similar to *x*-ray imaging, THz imaging will require high power and high resolution to advance relevant applications. Previously demonstrated THz imaging usually experiences one or several difficulties in insufficient source power, poor spectral tunability, or limited resolution from a low-wavelength source. A short-wavelength radiation source in the 5–10 THz is relatively scarce. Although a shorter wavelength improves imaging resolution, widely used imaging sensors, such as microbolometers, Schottky diodes, and photoconductive antennas, are usually not sensitive to detect radiation with frequencies above 5 THz. The radiation power of a high-frequency source becomes a key factor to realize low-noise and high-resolution imaging by using an ordinary pyroelectric detector. Here, we report a successful development of a fully coherent, tunable, > 100-kW-peak-power parametric source at 5.7 THz. It is then used together with a low-cost pyroelectric detector for demonstrating high-resolution 5.7-THz imaging in comparison with 2-THz imaging. To take advantage of the wavelength tunability of the source, we also report spectrally resolved imaging between 5.55 and 5.87 THz to reveal the spectroscopic characteristics and spatial distribution of a test drug, Aprovel.

## Introduction

THz radiation is known to penetrate a number of materials, such as plastics, polymers, and semiconductors, of which some are opaque to optical radiation. The relatively low frequency of a THz wave can also induce resonant absorption in large molecules in complex materials. As a result, THz imaging has become a powerful means to reveal hidden objects with high contrast in some materials^1,2^. This has been exploited in applications, such as security checking^3,4^, semiconductor quality assurance^5^, and others. Especially, tunable THz radiation is useful for wavelength-sensitive applications such as spectrally resolved imaging for illicit drugs^6^ and biomedical materials^7,8^. To be able to compete with *x*-ray imaging in systems, the THz source power, and its image resolution would still require a major upgrade. High source power is important to achieving enough signal-to-noise ratio, whereas the resolution of an image is scaled by the wavelength of the source illuminating the object. Currently, most THz imaging is performed with a source frequency below 2 THz^3,5,6,8^, where commercial microbolometer sensors, Schottky diodes, or photoconductive antennas are available for detection and image construction^9–11^. Although high-frequency THz radiation is advantageous for high-resolution imaging, low-cost image sensor arrays are not widely available in the 5–10 THz frequency range. On the other hand, bulk THz sensors, such as pyroelectric detector and Golay cell, which could be used in this frequency range, usually require an input energy of nJ–μJ to overcome the intrinsic background noise for detection.

In this work, we develop an ultra-high-power parametric source at about 6 THz for imaging by using KTP as the gain crystal. KTP is a nonlinear optical material having a peak parametric gain at 5.7 THz and it is discretely tunable between 1–8 THz and 11–13 THz via stimulated polariton scattering (SPS)^12–14^. In the SPS process, a laser pumps the nonlinear crystal to generate a THz wave and a red-shifted Stokes wave. Lithium niobate is also a popular nonlinear crystal for generating radiation with a peak gain around 2 THz^15–17^. In the high-frequency and high-power regime, KTP is a preferred material due to its broader SPS gain spectrum and higher laser damage threshold^18,19^. However, previously demonstrated THz KTP sources mostly adopted a pump pulse width of ~10 nanoseconds^14,20^. Such a long-pump-pulse scheme limits the pump field strength for efficient nonlinear frequency conversion before the material is damaged by the laser. Although we previously reported the generation of tens of kW peak power at 5.7 THz external to a KTP SPS source with an inferred ~ 0.1-ns pulse width^19^, the radiation wavelength of it could not be easily tuned for performing spectral imaging and its output power could not be easily maximized from the self-seeding scheme (see “Methods”). In this work, we employ a narrow-line tunable diode laser as the Stokes seed for ease of THz-wavelength tuning and further upgrade the peak output power to more than 100 kW with an experimentally verified pulse width. By using the high-power KTP parametric source and a bulk pyroelectric detector, we report in the following high-resolution raster-scan imaging at 5.7 THz. The resulting image is compared with that obtained from a similar 2-THz parametric source adopting lithium niobate as the gain crystal in the same setup. To take advantage of the tunable KTP THz source, we also report below spectrally resolved imaging for a test chemical, Aprovel, to reveal its spectroscopic characteristics with spatial resolution.

## Result and discussion

### High-power THz source

**Figure 1:**
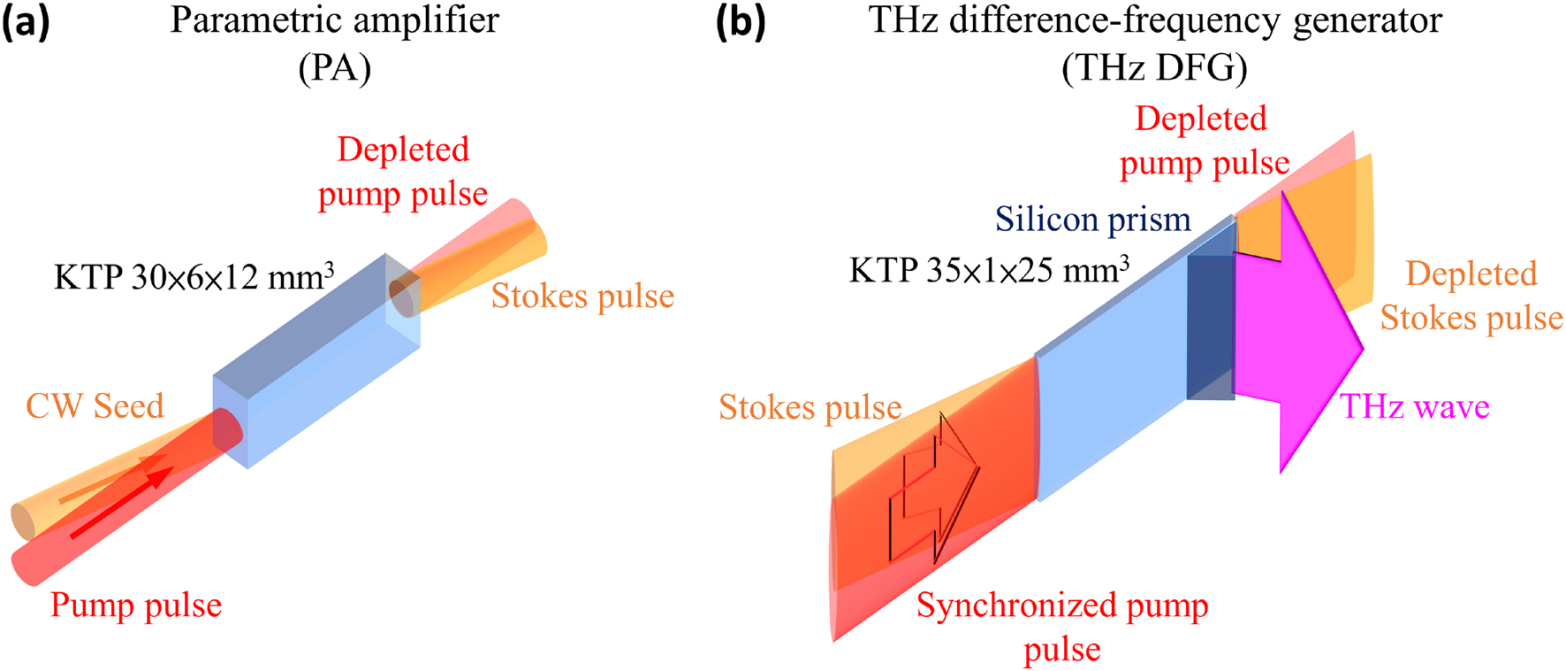
Schematic of the high-power THz parametric source using KTP as the gain crystal. The system consists of (**a**) a parametric amplifier (PA) for generating a strong Stokes pulse and (**b**) a subsequent difference frequency generator (DFG) for mixing the pump and Stokes pulses to generate high-power THz radiation. The pump pulses are generated by an amplified, passively Q-switched, single-longitudinal-mode laser at 1064 nm.

KTP is highly absorptive at THz frequencies. To maximize the THz output, it is desirable to perform difference-frequency generation in KTP and quickly couple out the THz wave. Figure 1 shows the two primary parts of our high-power THz parametric system using KTP as the gain crystal. The first part in Fig. 1a is a pulse-pumped parametric amplifier (PA) for generating a narrow-line red-shifted laser from a single-longitudinal-mode pump wave at 1064 nm. The red-shifted pulse, called the Stokes or signal pulse, is then injected into the second part of the system (Fig. 1b), which is a difference-frequency generator (DFG) producing a high-power THz pulse at the difference frequency of a pump pulse and the Stokes pulse. The pump pulses for both the PA and DFG are split from an amplified passively Q-switched Nd:YAG laser pulse with a single-longitudinal-mode and a 450-ps pulse width. The Nd:YAG laser system is capable of delivering more than 120-mJ pulse energy to the downstream PA and DFG. The seed for the PA is a continuous-wave (CW) external-cavity diode laser (ECDL) followed by a fiber laser amplifier. The ECDL is tunable between 1060 and 1090 nm, having a sub-MHz linewidth. The fiber laser amplifier boosts the ECDL seed power from a few mW up to 0.6 W. The nonlinear materials for the PA and THz DFG are optically polished uncoated KTP crystals, having dimensions of 30(*x*) × 6(*y*) × 12(*z*) and 35(*x*) × 1(*y*) × 25(*z*) mm, respectively. To have a high-energy throughput, the pump laser in the PA has an elliptical beam profile with radii of 2.1(*y*) × 2.8(*z*) mm along the minor and major axes, respectively. The signal-laser profile in the PA is circular, having a 2.8-mm radius to fully cover the elliptical pump beam and reduce amplified parametric fluorescence. In the THz DFG, both the pump and Stokes beam profiles are elliptical, having radii of 0.5(*y*) × 11.1(*z*) and 0.5(*y*) × 18(*z*) mm along the minor and major axes at the crystal center, respectively. The Stokes beam is again arranged to enclose the pump beam to avoid broadband optical parametric generation from the strong pump. The output beam profile of the THz radiation is therefore the overlapping area of the pump and Stokes beams in the DFG crystal. The thin crystal dimension along *y* permits off-axis oscillation for the THz wave and thus higher parametric efficiency for the SPS^21^. To utilize the largest nonlinear susceptibility *d*_33_ of KTP, all the mixing waves are polarized along the crystallographic* z* direction of KTP. A high-resistivity silicon prism is attached to the *y-*surface of the DFG KTP crystal to couple out the THz wave. A low-pass filter (LPF) with a cutoff wavelength at 20 μm is used to block the pump and Stokes from entering the pyroelectric detector at the output of the prism coupler.

**Figure 2:**
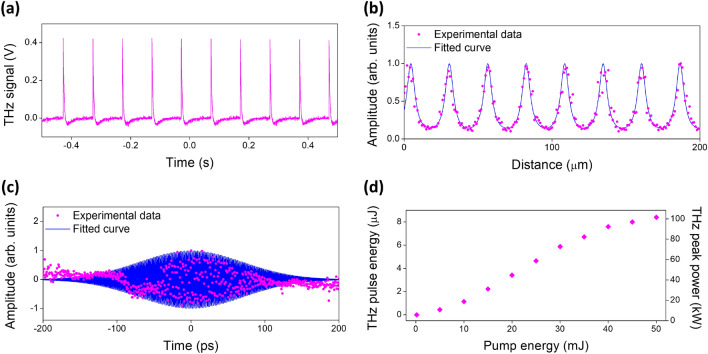
(**a**) The maximum THz-pulse signal detected by our pyroelectric detector (THz-20, SLT Sensor) when the Stokes energy and pump energy are 10 and 50 mJ in the DFG crystal, respectively. The corresponding THz pulse energy is 8.4 μJ. (**b**) Interference fringes scanned by a Fabry–Perot interferometer, indicating a THz wavelength of 52 μm. (**c**) Coarsely scanned Michaelson interferogram of the THz pulse fit by the autocorrelation of two Gaussian pulses with a wavelength of 52 μm, indicating a THz pulse width of 83 ps. (**d**) THz output energy (left vertical axis) and peak power (right vertical axis) versus pump energy. The Stokes pulse energy seeding into the THz DFG is fixed at 10 mJ. With the 83-ps pulse width deduced from (**b**), the maximum THz peak power detected by the pyroelectric detector is 100 kW.

In our experiment, we carefully verified the measured THz output energy by using two calibrated pyroelectric detectors, including one from SLT Sensor with responsivity of 0.175 V/μJ (THz-20 with a 1000X pre-amplifier) and the other from Gentec with responsivity of 4.03 V/μJ (THZ5I-BL-BNC)^22^. Figure 2a shows the maximum THz signal recorded by the THz-20 detector, when the pump and Stokes energy were 50 and 10 mJ, respectively, in the THz DFG. The transmittance of the low-pass filter in front of the pyroelectric detector is 28.6% at 5.7 THz. The peak signal amplitude of 420 mV in Fig. 2a therefore corresponds to a THz pulse energy of 8.4 μJ/pulse. The responsivity of the THZ5I-BL-BNC detector is about 23 times better and the same THz signal gave a peak amplitude of 9.7 V in the same measurement.

It is necessary to know the width of the THz pulse to determine the THz peak power. Since both the pump and Stokes waves are single-longitudinal-mode in the THz DFG, the generated THz wave is expected to have a transform-limited linewidth. To measure the THz wavelength, we first built a scanning Fabry–Perot spectrometer consisting of two reflecting plates made of high-resistivity silicon. Figure 2b shows the interference fringes of the THz pulse scanned by the Fabry–Perot spectrometer with a step size of 1 μm. The periodicity of the interference fringes confirms a 52-μm wavelength of the signal at 5.77 THz. To measure the THz pulse width, we further built a Michelson interferometer with a silicon beam splitter to measure the interferogram of the output THz pulse. The THz pulse width can be deduced from the envelope width of the interferogram. To avoid energy jitter over a long scan time, we did not intend to resolve individual fringes of the interferogram, but simply performed a coarse scan for the envelope by using a step size of 100 μm. Figure 2c shows the scanned envelope data fit by the autocorrelation of two Gaussian pulses with a wavelength of 52 μm and a pulse width of 83 ps. The Fabry–Perot spectrometer gives a better signal-to-noise ratio, because the data taking time is much shorter for just a few fringes. By using the 83-ps pulse width for the THz output, Fig. 2d plots the THz output energy (left vertical axis) and peak power (right vertical axis) versus pump energy. For this plot, the Stokes energy seeding into the THz DFG is fixed at 10 mJ. As can be seen from the figure, the maximum THz peak power emitted from the silicon prism exceeds 100 kW. Given the refractive index of 3.4 for silicon, the Fresnel loss on the air-side surface of the silicon prism is 30%. Internal to the silicon output coupler, the peak power of the THz radiation is estimated to be 142 kW, which could be mostly coupled out by using an anti-reflection layer atop the silicon^17^. The data in Fig. 2d does not show any obvious saturation even at the highest pump energy. Therefore, it is possible to further scale up the THz output power by increasing the pump and Stokes energy.

To compare, we list in Table 1 a few parameters between this work and two previously demonstrated high-power multi-cycle THz parametric sources using KTP. In 2020, Jia et al*.* reported the generation of 17-μJ pulse energy at 5.7 THz from KTP by using 580-mJ pump energy in a 7.5-ns width and 36.6-mJ Stokes energy^20^. If, similar to our case, the THz pulse width is approximately 5 times reduced from the pump or 1.5 ns, the corresponding THz peak power is 11.3 kW. Rubidium-doped KTP is often used for fabricating periodically poled KTP (PPKTP) for quasi-phase-matched nonlinear wavelength conversion^23^. In 2021, Tian et al*.* demonstrated the generation of 0.72-µJ output energy at 0.5 THz with a 3-GHz linewidth from a cryogenically cooled PPKTP by using a multi-line pump laser at about 1030 nm. Assuming a transform-limited pulse width for the 0.5-THz pulse, the corresponding THz peak power is about 5 kW. Refer to “Methods”. Our source is optimized to maximize the THz output power. The much higher peak power generated from this work benefits to applications requiring a high THz field.

**Table 1:** Comparison between multi-cycle THz parametric sources using KTP.

	This work	Jia et al.^20^	Tian et al.^23^
Material	KTP	KTP	Rb:PPKTP
Frequency	5.7 THz	5.7 THz	0.5 THz
Temperate	Room	Room	77 K
THz pulse energy (μJ)	8.4	17	0.72
THz peak power (kW)	101.2	11.3^a^	4.9^b^

^a^Assuming one-fifth of the pump pulse width.

^b^Assuming a transform limited pulse of 147 ps, although the pump pulse width is 250 ps.

### High-resolution THz imaging

**Figure 3:**
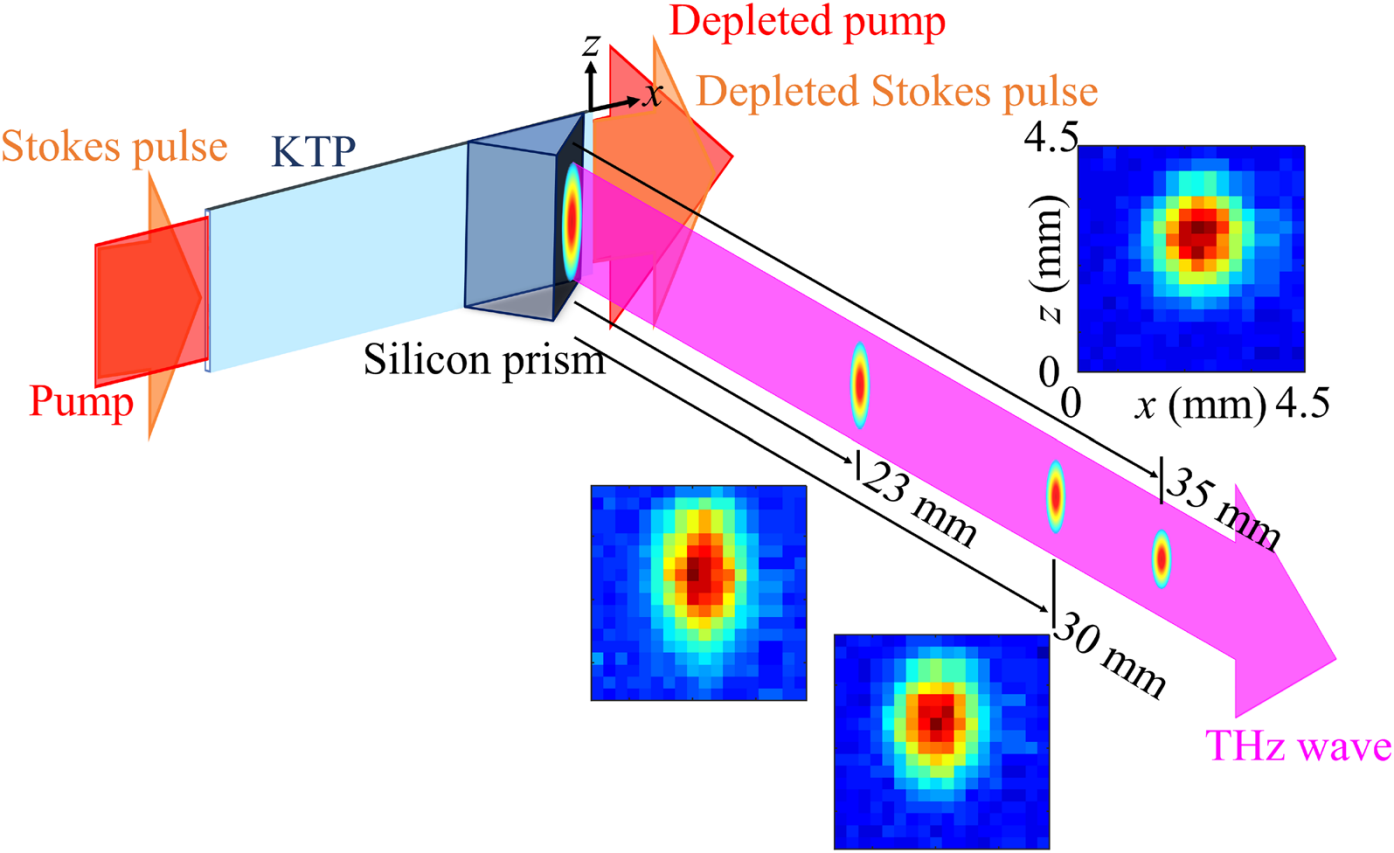
Scanning images of the 5.7-THz beam profile at 23, 30, 35 mm from the output of the silicon prism. The scanning step or the pixel size for the images is 250 μm. The THz beam is collimated in *x* and focused in *z* before the 35-mm location.

Imaging can be a power- or energy-demanding process, because the radiation signal collected on an image sensor is only a percentage of the total incident signal that is reflected from or transmitted through a sample. The excellent signal-to-noise ratio presented in Fig. 2a is promising for imaging using an ordinary bulk pyrodetector as a single-pixel detector. As a test, we mounted the THZ5I-BL-BNC pyroelectric detector on an *x*–*z* scanning stage to measure the THz beam profile through a 0.5-mm diameter aperture on the THz detector. Figure 3 shows the measured transverse profiles of the THz wave at different locations from the output of the silicon coupler. The scanning step, i.e., the pixel size, of the images is 250 μm. As can be seen from the images at 23, 30, 35 mm from the prism coupler, the THz beam is well collimated in* x* and focused in *z*, having a circular beam profile with a ~ 1 mm rms diameter at 35 mm from the prism.

In principle, the diffraction-limited resolution of an image is scaled by the wavelength of the light incident on the object. When compared with an ordinary source with a frequency below 2 THz, our ~ 6-THz source is expected to give 3-time better resolution for imaging. To compare, by using the same PA-DFG system, we replaced the KTP crystals with lithium niobate crystals to generate high-power radiation at 2 THz and performed an imaging test under the same experimental conditions. The ECDL wavelength was tuned to 1071 nm to match that of the Stokes pulse for lithium niobate in this case. The ultimate resolution of an imaging system is related to both the wavelength of the source and the numerical aperture of the system. In this comparison, we do not strive to build an imaging system with the highest resolution subject to a particular radiation wavelength, but intend to compare the quality of the images generated from the same imaging system with the 5.7 THz and 2 THz sources. Such a relative comparison avoids uncertainties from imaging systems with different numerical apertures.

**Figure 4:**
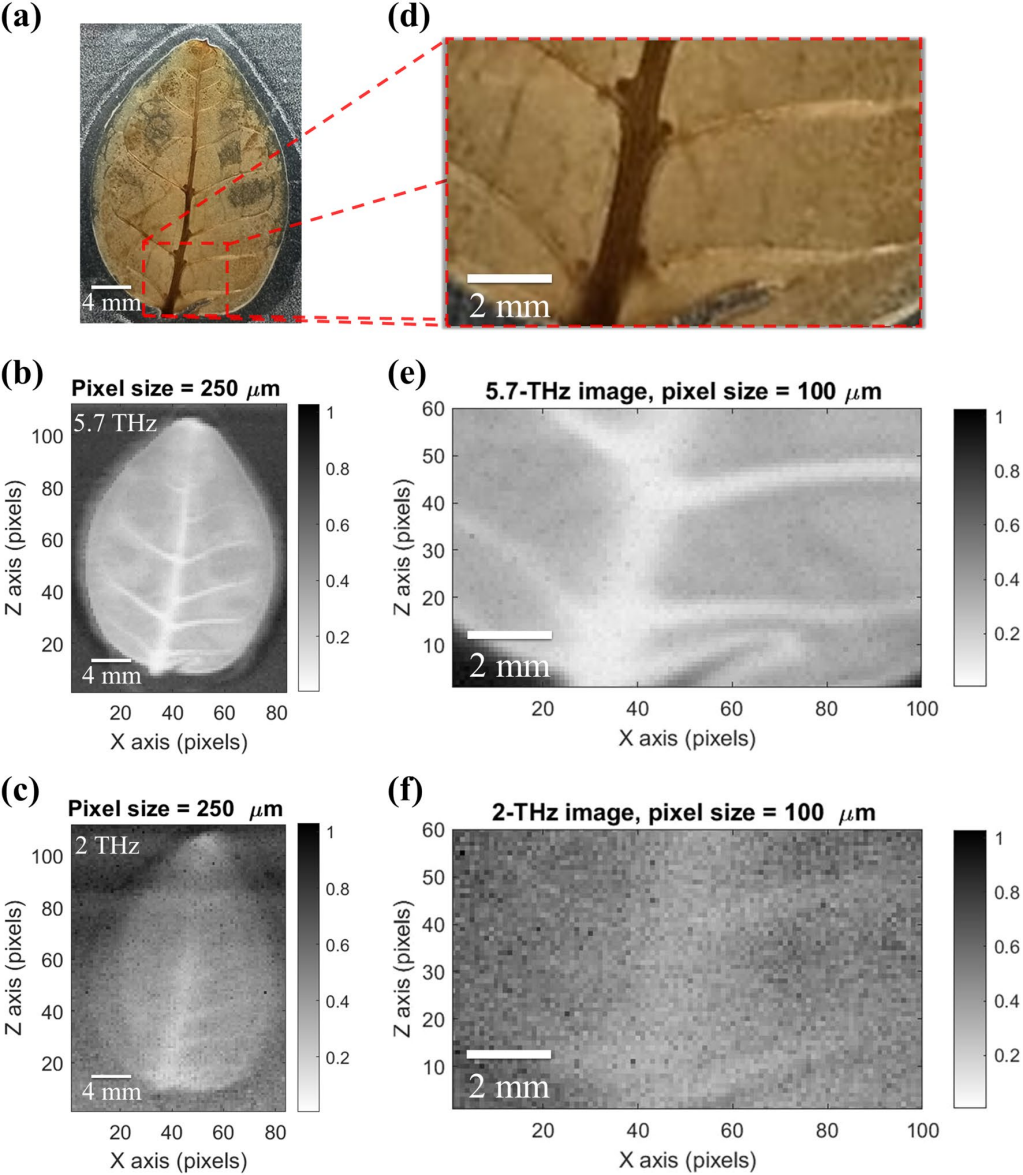
A dry leaf imaged at visible, ~ 5.7 THz, and ~ 2 THz. (**a**) Optical image of a leaf embedded between two polyethylene films. (**b**) 5.7-THz leaf image scanned with a step size of 250 μm. (**c**) 2-THz leaf image scanned with a step size of 250 μm. (**d**) Enlarged optical image of the leaf for the box area in (a). (**e**) 5.7-THz image of the boxed leaf scanned with a step size of 100 μm. (**c**) 2-THz image of the boxed leaf scanned with a step size of 100 μm. The resolution of the 5.7-THz images is apparently better.

The sample for imaging comparison at 5.7 and 2 THz is a leaf (Alternanthera bettzickiana (Regel) Nicholson)). Figure 4a shows the optical image of the leaf with its major and minor axes approximately equal to 25 and 20 mm, respectively. Figure 4b,c show the shadow images of the full leaf scanned by the focused 5.7 and 2 THz sources, respectively. The scanning step size or the pixel size is 250 μm. It is evident that the 5.7-THz image in Fig. 4b is much sharper than the 2-THz one in Fig. 4c. In particular, the primary veins of the leaf are clearly seen in Fig. 4b, but barely show up in Fig. 4c. To further improve the image quality, we continued to scan a small portion of the leaf with a 100-μm step size by using the 2 and 5.7 THz sources. Figures 4d–f show the optical, 5.7-THz, and 2-THz images of the boxed area of the leaf in Fig. 4a, respectively. The 5.7-THz image in Fig. 4e reveals the fine features of the veins and a tear at the right bottom corner of the leaf. On the contrary, the quality of the 2-THz image in Fig. 4f has little improvements, because the pixel size is already smaller than the imaging wavelength.

### Spectral imaging

**Figure 5:**
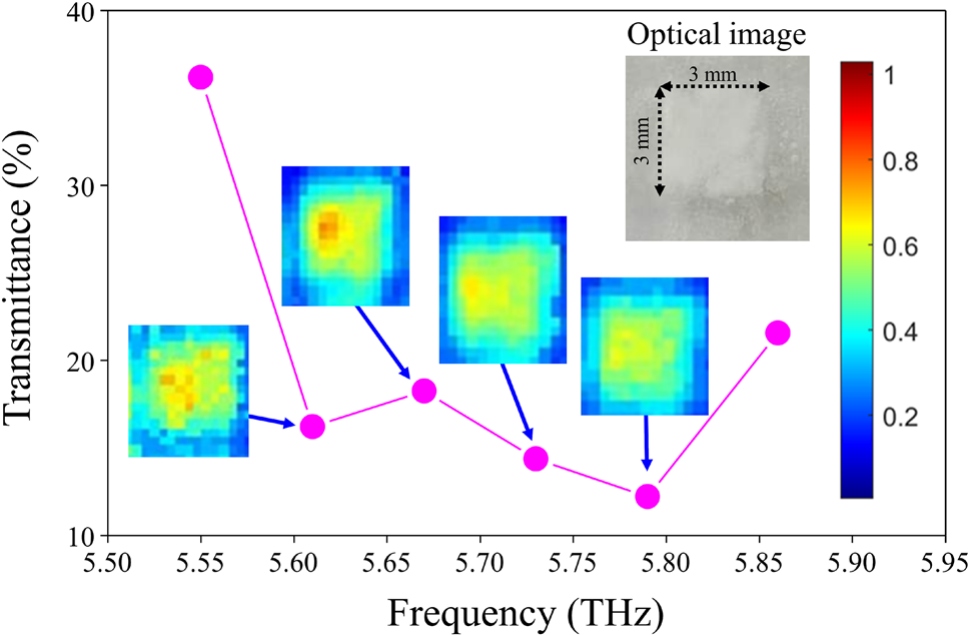
Spectral imaging of Aprovel powders. The characteristic transmittance curve of Aprovel powders between 5.55 and 5.87 THz. Insets: (false color) The THz transmittance images of the Aprovel powders between two polyethylene films scanned at 5.61, 5.67, 5.73, 5.79 THz, showing more transmission at 5.67 THz and less transmission at 5.79 THz over a spread area of 3 × 3 mm for the powders. (gray color) The optical image of the Aprovel sample between two polyethylene films.

A major advantage of the THz parametric source is its wavelength tunability. Popular THz parametric sources based on lithium niobate has two primary SPS gain peaks at 2 and 4.2 THz^15,24^, which limit the frequency tuning range to below 5 THz^25^. The angular bandwidth of the THz SPS in KTP can be a few hundred GHz. By varying the wavelength of the seeding ECDL, it becomes straightforward to tune the frequency of the KTP DFG between 5.5 and 5.9 THz. To demonstrate a spectrally resolved image, we select a popular drug for blood-pressure control, called Aprovel, as a test chemical. We first spread a small amount of Aprovel powder between two polyethylene films and measured its transmittance curve between 5.55 and 5.87 THz, as shown in Fig. 5. The tendency of the transmittance curve matches reasonably well to the known curve in the literature^26^. We then performed shadow imaging of it using the same imaging scanning system with a 250-μm pixel size at 4 frequencies, 5.61, 5.67, 5.73, 5.79 THz, and compare them (insets) with the characteristic transmittance curve. It is seen in Fig. 5 that the spectral images are indeed correlated with the variation of the transmittance curve. For instance, the image at 5.67 THz shows more transmission than the image at 5.7 THz. The false-color map also reveals the uneven distribution of the powders spread over an area of 3 × 3 mm (refer to the optical image in the inset). This experiment illustrates an opportunity for spectrally resolved imaging at high THz frequencies by using a tunable high-power coherent THz source and a bulk pyroelectric detector.

## Conclusions

In conclusion, we have developed an ultra-high-power, tunable, coherent, far-infrared radiation source at 5.7 THz based on pulsed difference frequency generation of two single-longitudinal-mode lasers in a thin KTP crystal. The measured maximum peak powers, external and internal to the silicon-prism output coupler atop the KTP DFG are 101 and 142 kW, respectively. The high peak power enables high-resolution scanning imaging at high THz frequencies by using a low-cost pyroelectric detector as a single-pixel image sensor. By using a leaf as a test sample, we show superior shadow images of it at 5.7 THz in comparison with those measured at 2 THz, under the same experimental conditions. By taking advantage of the wavelength tuning capability of the KTP high-power parametric source, we further demonstrated spectrally resolved images of a popular blood-pressure-control drug, called Aprovel, over a spread area of 3 × 3 mm. The experiment shows the potential to identify the spectroscopic content and the spatial distribution of chemicals using our tunable high-power and high-frequency THz source in conjunction with an ordinary pyroelectric detector.

## Methods

### THz source

**Figure 6:**
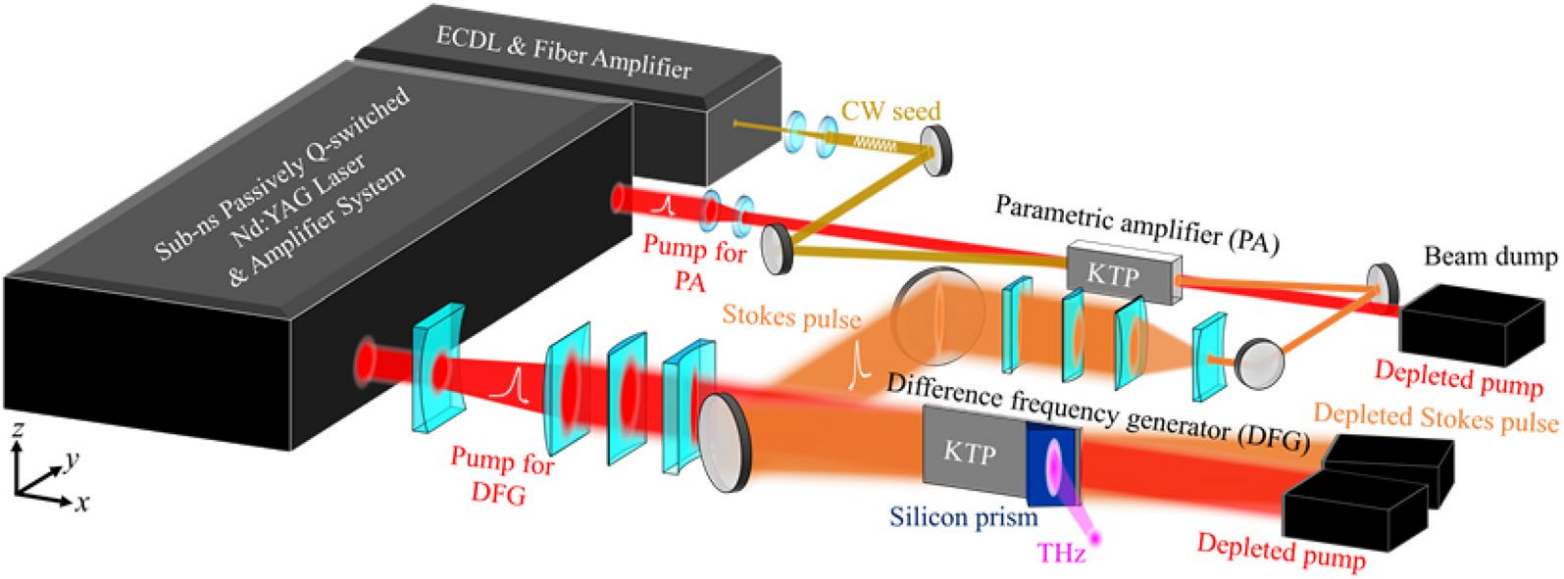
The schematic of the two-stage parametric source for demonstrating the high-resolution THz imaging. The first stage is a KTP PA seeded by a tunable diode laser, generating a high-power Stokes pulse. The second stage is a DFG for the pump and Stokes pulses, producing a high-power THz pulse via SPS in the second KTP crystal.

The coherent THz radiation is generated from the SPS in KTP and lithium niobate. An SPS process is the same as a parametric amplification process, except that SPS involves an enhanced parametric gain and absorption loss from Raman-like vibration of the material lattice. The peak SPS gains for KTP and lithium niobate are located at 5.7 and 2 THz, respectively. Figure 6 shows the schematic of our two-stage KTP parametric source for demonstrating the high-resolution THz imaging. The system is pumped by a sub-ns Nd:YAG laser amplifier at 1064 nm. The first stage is a KTP PA seeded by a tunable diode laser, generating a high-power red-shifted Stokes pulse. The second stage is a DFG for the pump and Stokes pulses, producing a high-power THz pulse via SPS in the 2^nd^ KTP crystal. A silicon prism is used to couple out the THz radiation from the 2nd KTP crystal for imaging experiments. Our system was optimized to generate as much THz power as possible from KTP. For SPS with high absorption at THz, the THz output is determined from the competition between the parametric gain coefficient Γ and the material absorption coefficient *α*_THz_, where Γ^2^ is proportional to the pump intensity. For KTP, *α*_THz_ = 252 cm^−1^ at 5.7 THz, which is significantly larger than the maximum parametric gain coefficient Γ = 15 cm^−1^ attainable at the maximum pump intensity of our laser system. In such a high-absorption and low-gain limit, the growth of the THz power *P*_*THz*_ is governed by the expression^27^

1 $$P_{THz} (L) = P_{s} \frac{{\omega_{THz} }}{{\omega_{s} }}\left( {\frac{2\Gamma }{{\alpha_{THz} }}} \right)^{2} (1 - e^{{ - \alpha_{THz} L/2}} )^{2}$$

where *P*_s_ is the power of the Stokes wave, *ω* is the angular frequency of the wave, and *L* is the crystal length. In our system, we first generate a pulsed *P*_s_ from a PA and inject it into a downstream DFG to generate *P*_THz_ according to Eq. (1). In our system, the PA and DFG are pumped by a single 120-mJ pulsed laser at 1064 nm. The pump pulse rate is 10 Hz. Assume that the total available power from the pump laser *P*_*0*_ is split into two parts, *rP*_0_ for pumping the PA and (1-*r*)*P*_0_ for pumping the DFG, where *r* is the power splitting ratio. The THz power in Eq. (1) is then proportional to the multiplication of the Stokes and pump powers in the DFG or

2 $$P_{THz} (L) \propto P_{s} P_{p} = \eta_{s} rP_{0} \times (1 - r)P_{0} ,$$

where *η*_s_ is the conversion efficiency for the PA and is typically saturated at ~ 15% at pump depletion^28^. The THz power in (2) is maximized when *r* = 50%. However, in our experiment, we chose to deliver more pump energy the PA to achieve the saturated efficiency. Specifically, a 70-mJ pump pulse is first injected into the PA to generate a ~ 10-mJ Stokes pulse, which is then mixed with the remaining 50-mJ pump pulse in the THz DFG to generate the highest THz output power in Fig. 2d.

### Scanning shadow imaging

**Figure 7:**
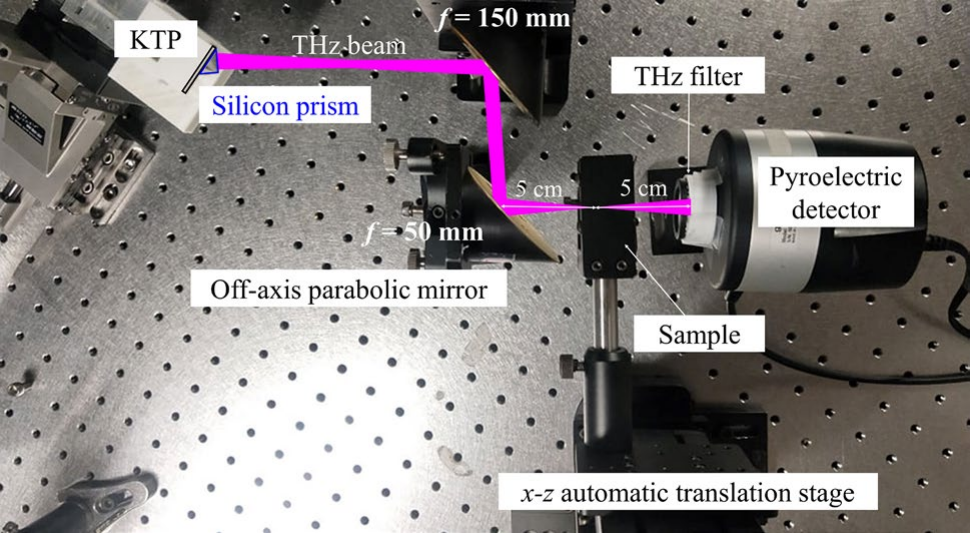
The experimental setup for performing scanning shadow imaging, wherein the THz wave is focused to the sample and the transmittance of it is recorded by a bulk pyroelectric detector.

Figure 7 shows the experimental setup for performing the shadow-imaging scan. The THz output is first collimated and focused to a focal point on a sample by a set of off-axis parabolic mirrors. The same PA-DFG system was installed with KTP and lithium niobate crystals to generate high-power 5.7- and 2-THz radiations for imaging, respectively. While transversely scanning the sample relative to the THz beam, we record the transmittance of the THz wave by using the THZ5I-BL-BNC detector behind the sample. The responsivity of the THZ5I-BL-BNC is 23 times better than the THz-20 detector. The sensor size of the detector is 5 × 5 mm, which is placed at 5 cm behind the sample. For each pixel data in the image, we used the built-in circuit of the oscilloscope to average 8 signal pulses from the incident THz wave.

**Figure 8:**
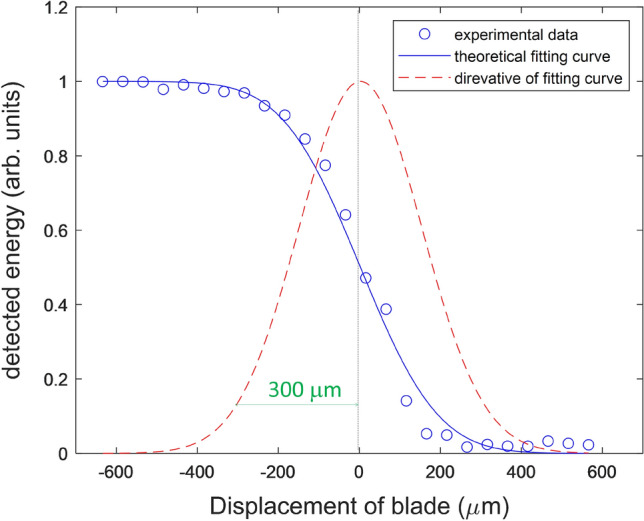
Knife-edge measurement of the focused 5.7-THz imaging beam. The blue line is the fitting curve of the experimental data (blue open dots) based an integrated Gaussian-intensity function. The red line is the derivative of the blue curve, indicating a Gaussian field radius of 300 μm.

To estimate the numerical aperture of our imaging system, we performed a knife-edge measurement at the focal point of the 5.6 THz beam. As shown in Fig. 8, the data points of the detected THz energy vs. knife-edge position (blue dots) are fitted by an integrated Gaussian-intensity curve (blue curve). By taking the derivative of the fitted curve, we plot in the same figure the Gaussian profile of the focused beam and a deduced field radius of *w*_0_ = 300 μm. The corresponding numerical aperture of the system is approximately the diffraction angle of the beam or NA ~ λ/π*w*_*0*_ = 0.056, where λ = 53 μm for the 5.7-THz wave.

**Figure 9:**
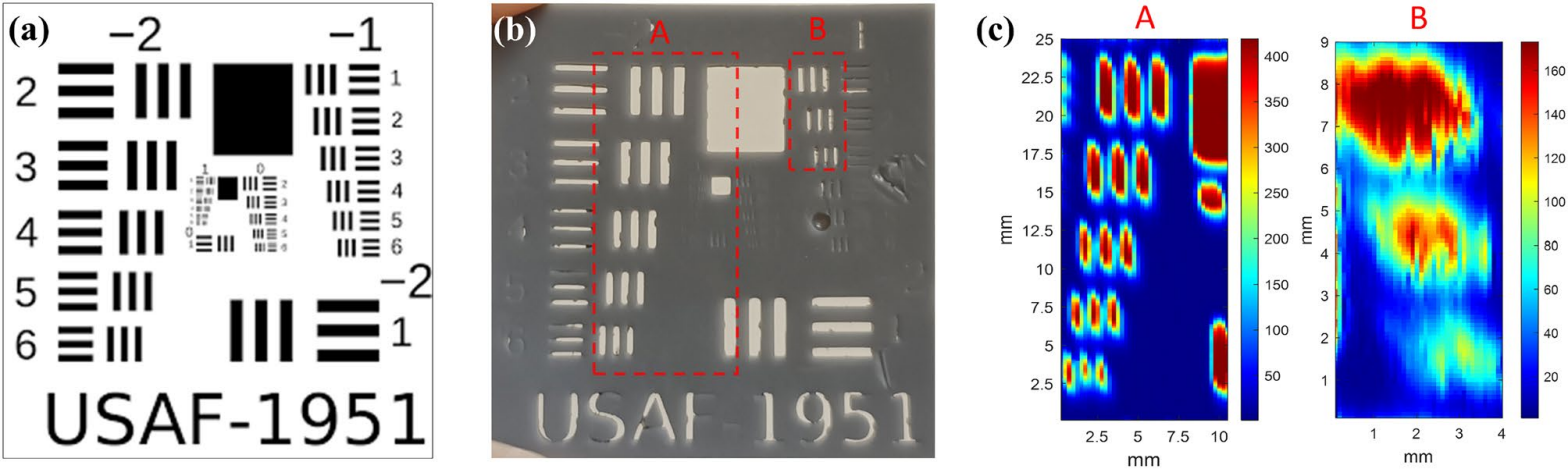
(**a**) The USAF-1951 test chart. (**b**) The optical image of the 3D-printed 3.4 cm × 3.4 cm USAF-1951 chart. (**c**) The 5.7-THz images of the boxed areas in (**b**). The least discernible bars have widths between 488 and 464 μm for Patterns − 2:6 and − 1:1, respectively.

To estimate the imaging resolution at 5.7 THz, we used a direct-light-processing (DLP) 3D printer (Zortrax Inkspire) to print out a 1-mm thick plastic USAF-1951 test chart and performed scanning shadow imaging for it. The resolution of the DLP printer is 50 μm. Figure 9 shows (a) the USAF-1951 chart, (b), the optical image of the 3D-printed 3.4 cm × 3.4 cm USAF-1951 chart, and (c) the 5.7-THz images of the boxed areas in (b). In Fig. 9c, the least discernible bars have widths between 488 and 464 μm for Patterns − 2:6 and − 1:1, respectively. This result is considered as the worst-case resolution for the 5.7-THz image, because the 50-μm resolution of the DLP 3D printing could be too large to reproduce the sharp edges of the test chart patterns. Although the system is not perfect for a 5.7-THz source, the side-by-side comparison in Fig. 4 unambiguously supports superiority of the images at 5.7 THz.

**Figure 10:**
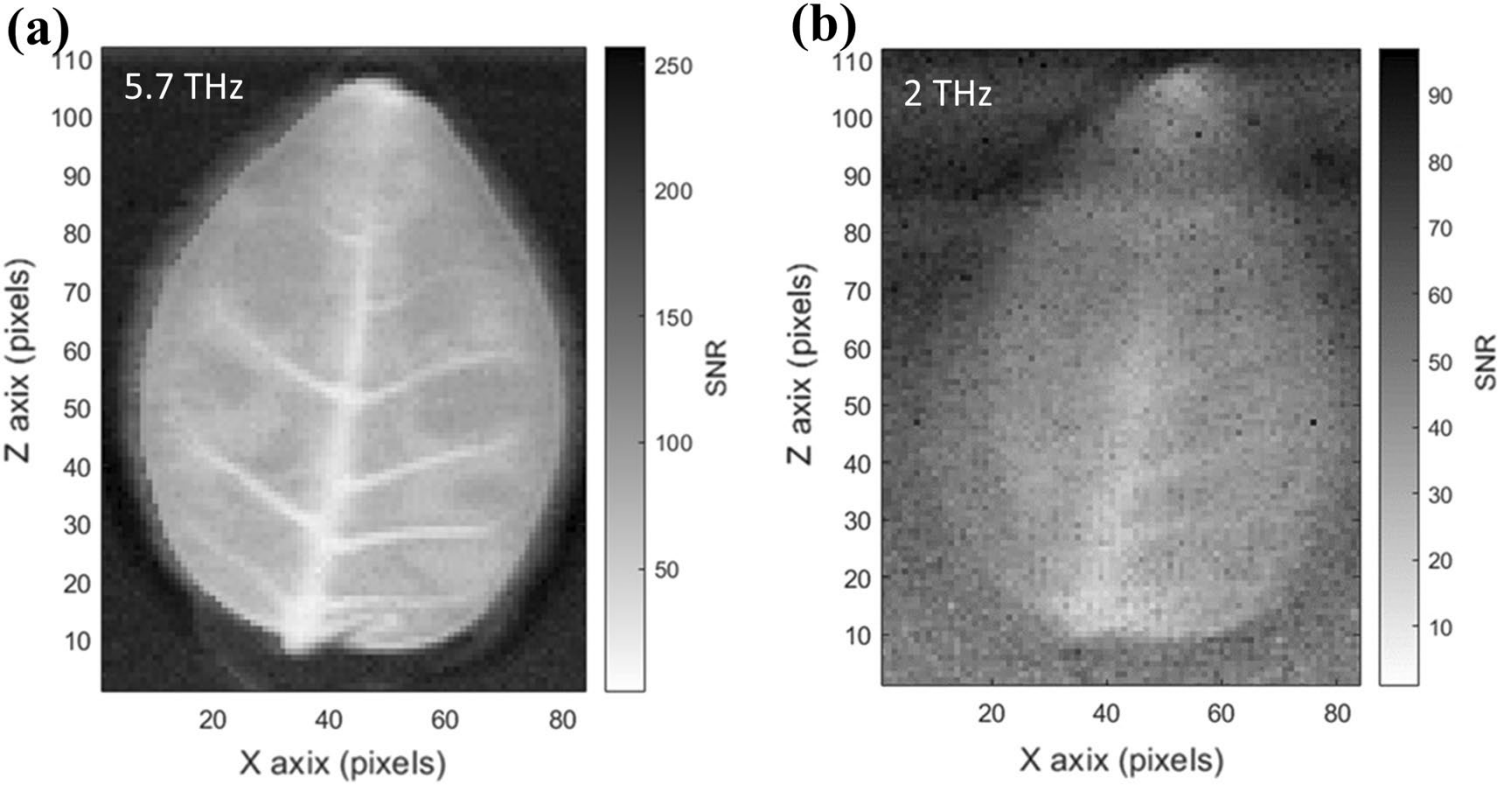
(**a**) SNR image of the leaf at 5.7 THz. Owing to the high material absorption, the leaf edge has relatively good contrast in the image. (**b**) SNR image of the leaf at 2 THz. Although the 2-THz source has a lower peak power, the best SNR in the image pixel is almost 20 dB.

When using the THZ5I-BL-BNC pyroelectric detector, we determined a noise floor of ~ 1 mV when setting 10-mV/scale for our oscilloscope. Figure 10 shows the shadow images of the leaf at (a) 5.7 and (b) 2 THz with each pixel’s gray scale proportional to the signal-to-noise ratio (SNR) in the detector. In (a), owing to the high material absorption, the image has better contrast at the leaf’s edge. Although the 2-THz source has a lower available peak power of about 6 kW for imaging, the best SNR in the image pixel is almost 20 dB.

### Sample preparation

The leaf was first freshly picked from a tree (Alternanthera bettzickiana (Regel) Nicholson)) and was embedded between two polyethylene films by using a commercial laminator. The thickness of the polyethylene film is 170 μm. The average transmittance of the leaf for the incidence wave at 5.7 THz increases from almost zero to about 40% when the color of the leaf slowly turns gray due to the loss of its water content. Once the THz transmittance of the leaf sample saturates at a fixed value, the same sample is used throughout the imaging experiments.

To perform spectral imaging, we first ground a few drugs, including Aprovel, LerZanidip, Aspirin, and Caffeine, and carefully distributed each kind of the powders into a 3 × 3 mm square area on a polyethylene film. We then covered the powders by using another polyethylene film and used the same laminator to fix the powders for transmittance measurements between 5.55 and 5.90 THz. We found that the THz transmittance curve of Aprovel has a relatively large variation in the frequency band of the measurement. We then proceeded with the spectral-imaging experiments for the 3 × 3 mm area of Aprovel to generate the images in Fig. 5.

## Data Availability

The data underlying the results presented in this work are available from the corresponding author upon request.
